# Very High Yield of Urgent Small-Bowel Capsule Endoscopy for Ongoing Overt Suspected Small-Bowel Bleeding Irrespective of the Usual Predictive Factors

**DOI:** 10.3390/diagnostics12112685

**Published:** 2022-11-04

**Authors:** Maria Manuela Estevinho, Rolando Pinho, Adélia Rodrigues, Ana Ponte, Edgar Afecto, João Correia, Teresa Freitas

**Affiliations:** 1 Department of Gastroenterology, Vila Nova de Gaia/Espinho Hospital Center, 4400-129 Vila Nova de Gaia, Portugal; 2 Unit of Pharmacology and Therapeutics, Department of Biomedicine, Faculty of Medicine, University of Porto, 4200-319 Porto, Portugal

**Keywords:** capsule endoscopy, emergency department, gastrointestinal bleeding, ongoing bleeding, small bowel bleeding

## Abstract

Evidence for an urgent approach to ongoing overt suspected small-bowel bleeding (SSBB) is scarce. We aimed to analyze our series of urgent small-bowel capsule endoscopies (SBCEs) for ongoing overt SSBB and to identify factors associated with positive findings and outcomes. A retrospective study of all SBCEs performed in the first 48 h after admission for overt SSBB between January 2006 and February 2022 was performed. Descriptive and inferential analyses (univariate and multivariable) were performed. Eighty-three urgent SBCEs were performed for overt SSBB. Patients were mostly men (69.2%, median age 68) and were followed for a median of 58.2 months (range 5–176). The diagnostic yield was 80.7%; in 60.2%, blood was detected in the small bowel (SB), while in 50.6%, a bleeding lesion was identified, mostly angioectasia. Patients with diabetes mellitus or taking NSAIDs were more prone to present SB findings, yet the explanatory power was low. Endoscopic or surgical treatments were performed in 28.9% and 19.3%, respectively, with the “non-conservative” therapeutic yield being 56.6%. Rebleeding occurred in 20.5% and was associated in the multivariable analysis with the female gender and anticoagulants use. This cohort of urgent SBCE, the largest from a European center, reinforces the usefulness of SBCE for ongoing overt SSBB management. This prompt performance of this procedure is highly effective, regardless of patients’ features.

## 1. Introduction

Gastrointestinal (GI) bleeding is associated with significant healthcare costs [1]. Even though the presence of melena has conventionally been associated with bleeding proximal to the ligament of Treitz, prompting the use of esophagogastroduodenoscopy as the first diagnostic procedure, this clinical sign has modest localization value [2,3]. Moreover, recent studies have suggested a relative decrease in the incidence of upper GI bleeding [2].

Traditionally, obscure gastrointestinal bleeding (OGIB) was defined as bleeding of unknown origin that persisted or recurred after negative upper endoscopy and ileocolonoscopy. More recently, it was proposed that such a term should be reserved for patients in whom the source could not be identified also after small-bowel (SB) endoscopic and/or radiographic imaging, as these modalities detect the bleeding cause in up to 75% of patients [4].

SBCE has a key role in the diagnosis of suspected small-bowel bleeding (SSBB); it is widely available and has a good safety profile. In addition, the early performance of SBCE has been reported to translate into better patient management [5,6]. Accordingly, the European Society of Gastrointestinal Endoscopy recommends performing SBCE as soon as possible after the bleeding episode, although a timespan of up to 14 days is recommended. Even though the recent metanalyses advise an earlier approach, tendentiously within the first 48 hours [6], the optimal timepoint remains to be determined. The management of mid-GI bleeding may be challenging, as it relies on a sequence of endoscopic procedures that require time and resources. Even though several studies in the setting of iron-deficient anemia have identified several factors associated with a higher probability of detecting positive findings in elective SBCE [7,8,9], such as being male, being older than 65 years, and having heart failure or chronic kidney disease, no predictors have been identified in the setting of ongoing overt bleeding. Therefore, we intended to analyze the experience of our tertiary referral center with SBCE performed in an urgent setting (up to 48 h after admission), during ongoing overt SSBB and to study patient-related factors that could be useful to predict positive SBCE findings and patients’ prognosis.

## 2. Materials and Methods

### 2.1. Study Definitions and Endpoints

Suspected small-bowel bleeding (SSBB) was defined as recurrent or persistent gastrointestinal bleeding after negative esophagogastroduodenoscopy (EGD) and ileocolonoscopy. Ongoing overt SSBB was defined as visible bleeding (melena or hematochezia) at the time of endoscopic evaluation (including SBCE). Urgent SBCE was defined as SBCE performed within 48 h of hospital admission related to the bleeding event and during ongoing overt SSBB. SBCE findings were classified according to standard practice [10] as lesions with high bleeding potential (P2) and those with uncertain or no bleeding potential (P1 and P0, respectively). Angioectasia, Dieulafoy’s lesion, arteriovenous malformation, varix, tumor, polyp, ulceration, diverticulum, or the presence of blood and/or blood clots in the lumen of the SB were classified as P2 lesions, as previously described [6]. The primary endpoints were the diagnostic and “non-conservative” therapeutic yields. The diagnostic yield was defined as the proportion of patients presenting blood or at least one P2 lesion [10] in the SB, and/or presenting bleeding lesions or blood outside the SB. Endoscopic, angiographic, and surgical treatments were considered to calculate the “non-conservative” therapeutic yield (proportion of patients in whom at least one of those treatments was carried out). The secondary endpoints were rebleeding and mortality. Rebleeding was defined as a documented fall in hemoglobin of 2 g/dL from baseline, evidence of melena or hematochezia, or need for blood transfusion [11,12]. Mortality during follow-up, associated or not with gastrointestinal bleeding, was registered.

### 2.2. Patients’ Selection and Data Collection

A retrospective cohort of patients with overt SSBB who underwent SBCE in the first 48 h after hospital admission, promptly after bidirectional endoscopy, from January 2005 to February 2022 at the Vila Nova de Gaia Espinho Hospital Centre, was evaluated. Exclusion criteria were being younger than 18 years, not having prior EGD and ileocolonoscopy, and/or being lost to follow-up. Patients were followed-up until death or up to July 2022. Patient clinical information was retrospectively collected from electronic medical records, including demographic characteristics (gender and age), comorbidities (chronic heart, kidney or liver disease, diabetes mellitus, previous abdominal surgeries, and malignant neoplasm), current medication (anticoagulants, antiplatelets, nonsteroidal anti-inflammatory drugs (NSAIDs), and proton pump inhibitors), symptoms/signs at admission to the emergency department (melena, hematochezia, and altered mental status), blood pressure and heart frequency and analytical parameters at admission (hemoglobin (Hg), international normalized ratio (INR), platelets, serum creatinine (sCr), and blood urea nitrogen (BUN)), and units of packed red blood cells (RBCs) transfused prior to SBCE. Informed consent was signed by all patients prior to each endoscopic procedure. The study protocol conforms to the ethical guidelines of the 1975 Declaration of Helsinki and was approved by the ethical review board of Centro Hospitalar de Vila Nova de Gaia Espinho. 

### 2.3. Capsule Endoscopy Procedure

SBCE was performed using Pillcam SB/SB2 (Medtronic) or Mirocam MC1200/1600 (Intromedic), depending on the availability. Considering the urgent setting and that patients had performed colonoscopy immediately before swallowing SBCE, no additional bowel preparation was required. A prokinetic agent (metoclopramide 10 mg) was administered if the SBCE was retained in the stomach after 1–2 h of ingestion. The recorder was removed after 12 h after capsule ingestion, or earlier if the real-time view confirmed that the device had reached the colon (complete procedure). The videos were analyzed by gastroenterologists with expertise in SBCE (each with over 350 SBCE procedures), at maximum speeds of 10 and 12 frames per second for Pillcam and Mirocam, respectively. The reports were evaluated to collect findings and other procedure-related data (completeness and bowel preparation (according to the Brotz scale [13], with a score ≥7/10 being adequate mucosal visualization)). Data on further endoscopic management after SBCE, including endoscopic, angiographic, and surgical treatment, new bleeding episodes, and/or death (related or not with the SSBB episode), were also retrieved.

### 2.4. Statistical Analysis

In this retrospective cohort study, both descriptive and inferential statistics were used. The sample size was calculated for the two main endpoints (diagnostic and “non-conservative” therapeutic yields) as previously reported [14]; considering data from prior studies, we determined that at least 72 patients were needed to detect significant differences on two-tailed tests with a 0.05 alpha level and 80% power. Categorical variables were presented as frequencies and percentages. Continuous variables were presented as mean and standard deviation (when the distribution was normal), or as median and interquartile range (IQR, quartile 1–quartile 3) for a skewed distribution. Categorical data were compared using the chi-square test. For continuous data, the student’s T test was used. A backward stepwise Cox Regression was used for multivariable analysis. The Nagelkerke’s R squared [15] was used to estimate the predictive ability of the multivariable models; this metric ranges between 0 and 1 and corresponds to the proportion of total variance explained. The Kaplan–Meier method was used to analyze the survival time and the time to rebleeding; the log-rank test was applied to compare groups. Statistical analysis was performed using the SPSS Statistics software (version 28, IBM Inc. Armonk, NY, USA). All reported *p*-values were two-tailed, with a *p*-value below 0.05 indicating statistical significance.

## 3. Results

### 3.1. Demographic and Clinical Characteristics of the Cohort

Between January 2005 and February 2022, 83 SBCEs for ongoing overt SSBB were performed in our endoscopy unit; all patients were hospitalized. The baseline characteristics of the cohort are described in Table 1. Patients had a median age of 68.0-years-old (ranging from 21 to 90) and were mostly male (69.2%). Most of the patients presented with melena (57.8%), while the remaining presented hematochezia. Regarding comorbidities and medication, 37.3% of the patients had chronic heart disease, 33.7% had diabetes mellitus, and 15.7% had chronic kidney disease. Concerning medication, 39.8% were taking NSAIDs and 30.1% were under anticoagulants. Most of the patients required transfusion prior to SBCE (n = 51, 61.4%), with median transfusion requirements of 2.3 (IQR 0.0–2.0). The median follow-up time was 57.9 (IQR 18.0–96.0), ranging from 5 to 176 months.

### 3.2. SBCE Findings

The SBCE procedure was classified as incomplete in 15 out of 83 patients (18.1%), as the device did not reach the cecum (Supplementary Table S1). The only patient-related factor associated with incomplete SB examination was having diabetes mellitus (*p* = 0.032) (Supplementary Table S2). Complete SB examination was not associated with higher diagnostic yield (*p* = 0.584), nor with the detection of vascular lesions (*p* = 0.281). SB cleansing was classified as inadequate (below 7 points in the Brotz scale [13]) in 17 procedures; yet, this did not impact the detection of lesions (*p* > 0.050). Inadequate cleansing was more common in patients above 65 years old (*p* = 0.046).

**Figure 1:**
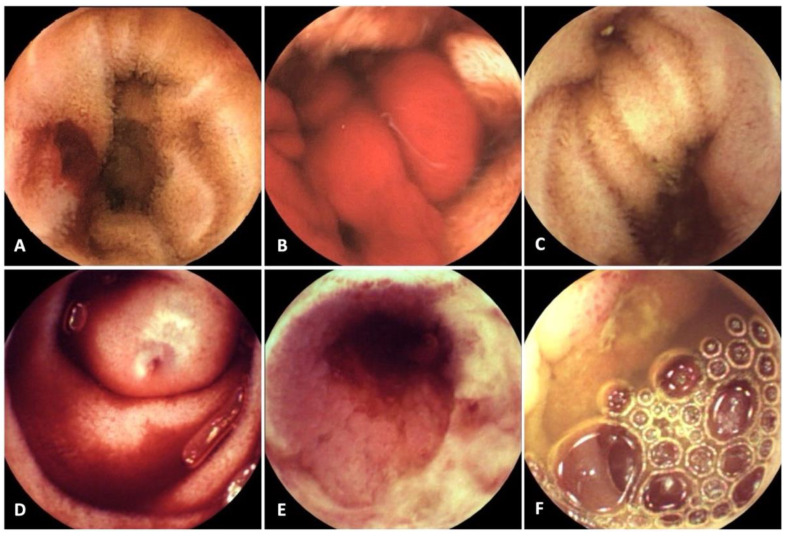
Examples of positive findings in capsule endoscopy—(**A**) bleeding jejunal angioectasia; (**B**) blood without identifiable bleeding lesion; (**C**) Meckel diverticulum with erosion; (**D**) ileal varix with rupture point; (**E**) ulcerated ileal stenosis; (**F**) jejunal ulcer.

Overall, 67 patients (80.7%) presented blood or at least one lesion with bleeding potential in the SB or in other gastrointestinal locations amenable to SBCE detection (Table 1)–this percentage corresponded to the diagnostic yield. Positive findings in the SB were detected in 62 patients (74.7%); a bleeding lesion was recognized in 42, while only blood (no perceivable lesion) was identified in 20. The most common lesions in the SB were angioectasia (n = 15), ulcers (n = 10), or neoplastic lesions (n = 6). Some examples of these lesions are depicted in Figure 1.

The presence of blood or lesions was more frequent in the ileum (n = 34, 54.8%), while only 4 patients had findings in the duodenum, distal to the ampulla of Vater. The comparison of patient-related characteristics depending on the presence or absence of findings is presented in Table 2.

The only factors found to be significantly associated with higher diagnostic yield and with the presence of lesions in the SB were being diagnosed with diabetes mellitus (*p* = 0.004 and *p* = 0.031, respectively) and taking NSAIDs (*p* = 0.049 and *p* = 0.047, respectively). On the other hand, other comorbidities, taking anticoagulants or antiaggregant drugs, the heart rate and blood pressure at admission, analytical parameters, or transfusion requirements were not statistically relevant. Vascular lesions were significantly more prevalent in patients with chronic kidney disease (CKD) (*p* = 0.046) and who had melena at hospital admission (*p* = 0.042). 

In line with these findings, the multivariable analysis (Table 3) corroborated that having diabetes was significantly associated with higher odds of having lesions or blood detected by SBCEs (OR 8.817, *p* = 0.002) and in the SB (OR 10.666, *p* = 0.012), yet taking NSAIDs was not an independent predictor.

The Nagelkerke’s R squared for these multivariable models was 0.233 and 0.225, respectively. Concerning vascular lesions’ detection, presenting with melena, or having known kidney disease were identified as independent predictors on the multivariable analysis (OR 4.007 (*p* = 0.044) and 4.944 (*p* = 0.029), respectively). Lesions or blood outside the SB were detected in 15 patients (18.1%), namely colonic angiodysplasia (n = 4), duodenal ulcer (n = 2), Dieulafoy’s lesion of the duodenum (n = 3), bleeding colonic polyp (n = 2), and diverticular bleeding (n = 4). Diagnosis outside the SB was more frequent in patients with a heart rate/systolic blood pressure ratio at admission below 1 (41.2% vs. 15.2%, *p* = 0.018 (Table 2)); this factor was also significant in the multivariable analysis (OR 3.436, *p* = 0.045, Table 3).

### 3.3. Therapeutic Yield

**Figure 2:**
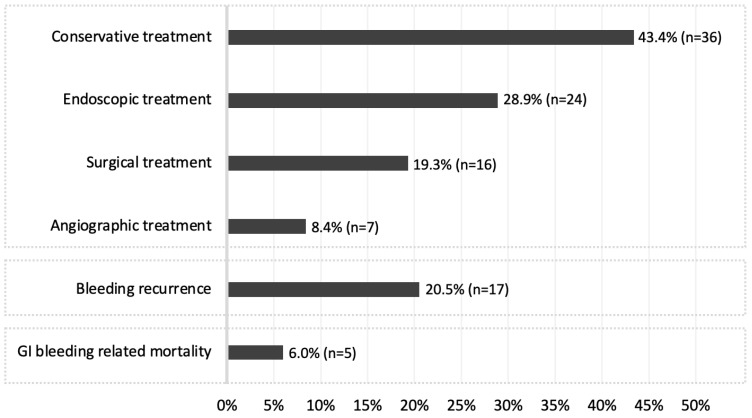
Outcomes after SBCE. Gastrointestinal (GI).

In this cohort, 43.4% of the bleeding episodes were managed conservatively (clinical monitoring with or without suspension of antiplatelet or anticoagulant drugs, blood transfusions, and/or iron supplementation) (Figure 2).

In 28.9% of the patients (n = 24), endoscopic treatment was performed, mostly argon plasma coagulation (50.0%) or adrenalin injection plus clips placement (12.5%). The remaining were submitted to surgery (19.3%) or to angiographic treatment (8.4%). Patients requiring nonconservative treatment were mostly women (72.7% vs. 48.0%, *p* = 0.026), while no significant differences were found for other clinical characteristics (Table 4).

In line with this, in the multivariable analysis, males had an OR for requiring therapeutics of 0.346 (*p* = 0.028), yet the explanatory power of this nominal variable was low (Nagelkerke’s R squared = 0.090) (Table 3). SB cleansing or incomplete SB visualization did not impact the need for nonconservative therapeutics or the tendency for rebleeding (Table 3).

### 3.4. Rebleeding

Rebleeding occurred in 17 patients (20.5%, Figure 2), after a median of 2.0 months (IQR 1.5–31.5; ranging from 1.0 to 82.0). Most rebleeding occurred in the first 5 months after the index hemorrhage (64.7%). Rebleeding at 6, 24, and 36 months was 13.3%, 14.5%, and 19.9%, respectively. Notably, only 29.4% of the patients presenting rebleeding had been managed conservatively in the first bleeding episode. The etiologies of patients who rebled were mostly angioectasia (70.6%) or tumors that had already been recognized in the first bleeding episode and that were pending surgical removal (17.6%, all confirmed as gastrointestinal stromal tumors in the enterectomy specimens). Patients who rebled were mostly women (39.4% vs. 8.0%, *p* < 0.001), or chronically under anticoagulants (*p* = 0.029) or antiaggregant drugs (*p* = 0.031) (Table 3). The first two variables were independent predictors in the multivariable analysis (Table 4); the combination of the three variables allowed the explanation of 29.8% of the outcome (Nagelkerke’s R squared = 0.298). However, the time to rebleed was not statistically different between genders neither among users of anticoagulant nor antiaggregant drugs.

### 3.5. Mortality

During follow-up, up to July 2022, 27 patients died (32.5%), with a mortality rate of 9.6%, 16.9%, and 22.0% at 6, 24, and 36 months, respectively. Five deaths were directly related to the gastrointestinal bleeding (Figure 2); two of these occurred up to one week after the bleeding episode; the remaining occurred after 3, 7, and 11 months and in the setting of rebleeding. The only factors significantly associated with overall mortality were being older than 65-years-old (*p* = 0.043) and having CKD (*p* = 0.023), although not associated with time to mortality. On the other hand, none of the evaluated patient-related factors were significantly associated with bleeding-related mortality.

## 4. Discussion

This study analyzed the performance of SBCEs in the urgent setting (up to 48 h after hospital admission) in patients with ongoing overt SSBB. As far as the authors know, and based on a recent metanalysis [6], this is the largest European cohort of urgent SBCE and the first with such a long follow-up (median of almost 5 years). To date, 16 studies reported the performance of SBCE for ongoing bleeding or performed up to 48 h after admission to the emergency department. In all these studies, apart from one [16], the number of patients were between 11 and 45. Interestingly, the largest study [16], which was performed in India and included more than 100 patients, was published more than 10 years ago. In addition to that, our study was powered to identify predictive factors for the two primary endpoints (detecting positive findings and need for therapy). In our series of 83 patients, no cases of retention nor adverse events occurred, corroborating the safety of SBCE. However, 18.1% of the SB examinations were incomplete. This percentage is higher than that registered in elective SBCE (10.1% in a recent series of almost 2000 SB examinations) [17]. This discrepancy may be related to the hospitalization status and consequent reduced mobility, and to the relatively high frequency of diabetes mellitus in our cohort (around one third of the patients; identified as significant predictor in the univariate analysis). These features had already been identified as potential risk factors for incomplete SB visualization [18,19]. Inadequate cleanliness was registered in 20.5% and found to be associated with older age; this percentage may be due to the lack of additional purgatives (all patients had only drunk bowel preparation before colonoscopy). Indeed, at present, no recommendation exists regarding SB preparation when SBCE is performed promptly after colonoscopy [20].

Even though previous studies on elective SBCE have highlighted that incomplete SB visualization and inadequate cleansing could potentially decrease the diagnostic yield and predispose to delayed intervention [21,22], our diagnostic and “non-conservative” therapeutic yields seemed unaffected. Overall, the diagnostic yield was 80.7%, with it being possible to identify a bleeding SB lesion in 84.0% of these patients, mostly angioectasia (35.7%). Our results are in line with the literature [6]—pooled diagnostic yield of 81.3% for overt SSBB, with vascular lesions being the most prevalent [23]. It is important to acknowledge that this diagnostic yield is much higher than that registered for obscure SSBB, which ranges between 40.0 and 50.0% [24,25].

The multivariable analysis identified some factors associated with the presence of positive findings inside the SB (having diabetes mellitus or taking NSAIDs), and in other regions amenable to SBCE visualization (having CKD or presenting a heart rate/systolic blood pressure (SBP) ratio below 1 at admission). Having diabetes mellitus and taking NSAIDs had already been associated with a higher chance of presenting SB lesions [26,27]. The higher risk for lesions outside the SB (angioectasia, peptic ulcers, esophagitis, diverticular bleeding, and ischemic colitis) had already been reported in patients with CKD [28]. The fact that patients with non-SB findings had a lower heart rate/SBP ratio was expected, as hemodynamic instability is more common in non-SB bleeding, particularly proximal to the ligament of Treitz [29].

In our cohort, most of the patients were managed non-conservatively, mainly endoscopically (28.9%), with the “non-conservative” therapeutic yield being 56.6%. This value is similar to that pooled from six previous studies in which SBCE was also performed in the first 48 h (59.1% [6]).

Rebleeding was registered in 20.5% after a median follow-up of 57.9 months, mostly due to angioectasia (more than 70%). Patients taking anticoagulants had almost four-times-higher odds of rebleeding. Incomplete SB examination or poor preparation was not identified as a rebleeding predictor. In addition, being treated conservatively did not increase rebleeding. Only two prior studies on SBCE in the first 48 h evaluated rebleeding, with rates of 18.5% [30] and 11.0% [31] at 36 months. Higher rebleeding percentages were reported in the setting of obscure SSBB, with values ranging between 38.5% [32] (at 24 months) and 50% [33] (at 60 months).

Overall, 27 patients (32.5%) died during follow-up, which was longer than 8 years for almost one third of the cohort. This is in line with what has been previously reported [30,34] and was expected considering patients’ age and comorbidities. It should be noted that only five died directly from bleeding-related causes.

The analysis of our series allows the conclusion that: (i) the diagnostic and “non-conservative” therapeutic yields of SBCE performed for ongoing overt SSBB in the first 48 h after admission are very high, particularly in comparison to those reported for obscure SSBB, supporting the key role of this procedure in the urgent management of these patients; (ii) the multivariable models using patient-related characteristics were poor-to-modest in explaining the detection of blood or lesions, the need for non-conservative therapeutics, and the occurrence of rebleeding. Therefore, it may be hypothesized that in the urgent setting, time may be the most important factor influencing SBCE performance and patients’ outcomes. Accordingly, instead of selecting for urgent SBCE the patients that are more prone to present findings, the priority should be to perform SBCE in all patients during the first 48 h after admission. Despite describing a large cohort, being powered for the two main endpoints, and having a long-follow up, this study has some limitations, including the single-center and retrospective design. Prospective cost-effectiveness analyses are required to further analyze the impact of early SBCE in this group of patients. In addition, comparative studies may uncover the patients that may benefit from even earlier SBCE, challenging the algorithm and performing SBCE before colonoscopy.

## Figures and Tables

**Table 1:** Baseline characteristics of the cohort performing small-bowel capsule endoscopy (SBCE) in the first 48 h after hospital admission and SBCE findings.

Cohort Characteristics	
Age—median (IQR)	68.0 (58.0–77.0)
Male—% (n)	69.2% (50)
Presenting with melena—% (n)	57.8% (48)
Chronic heart disease—% (n)	37.3% (31)
Diabetes mellitus—% (n)	33.7% (28)
Chronic kidney disease—% (n)	15.7% (13)
Chronic liver disease—% (n)	8.4% (7)
Malignant neoplasm—% (n)	6.0% (5)
Patients taking anticoagulants—% (n)	30.1% (25)
Patients taking NSAIDs—% (n)	39.8% (33)
Systolic blood pressure at admission—median (IQR)	116.0 (100.0–137.0)
Heart rate at admission—median (IQR)	88.0 (78.0–95.0)
Haemoglobin at admission—median (IQR)	8.2 (6.7–10.1)
International normalized ratio—median (IQR)	1.1 (1.0–1.7)
Blood urea nitrogen/creatinine ratio—median (IQR)	30.6 (21.6–40.3)
Transfusion requirements prior to SCBE—median (IQR)	2.3 (0.0–2.0)
Follow-up (months)—median (IQR)	58.2 (18.0–96.0)
SBCE findings—% (n)	
Presence of blood in the small bowel	60.2% (50/83)
Lesion with bleeding potential in the small bowel (P2)	50.6% (42/83)
Angioectasia	35.7% (15/42)
Hemangioma	4.8% (2/42)
Ulcer	23.8% (10/42)
Epithelial neoplasm (adenocarcinoma n = 2, neuroendocrine tumor n = 4)	14.3% (6/42)
Sub-epithelial neoplasm (GIST n = 5)	11.9% (5/42)
Varix	2.4% (1/42)
Diverticulum	7.1% (3/42)
Lesion or blood outside the small bowel	18.1% (15/83)

Interquartile range (IQR); non-steroidal anti-inflammatory drugs (NSAIDs); number (n); percentage (%).

**Table 2:** Comparison of characteristics among patients with positive findings in small-bowel capsule endoscopy (SBCE), submitted to endoscopic or surgical therapy, and presenting rebleeding (univariate analysis).

Baseline Characteristics		Positive Findings (n = 67)		Findings in the SB (n = 62)		Vascular Lesion (n = 16)		Findings Outside the SB (n = 15)	
Male sex	Yes	41/50	*p* = 0.745	36/50	*p* = 0.334	9/50	*p* = 0.717	10/50	*p* = 0.673
	No	26/33		26/33		7/33		5/33	
Presenting with melena	Yes	38/46	*p* = 0.874	36/46	*p* = 0.281	12/46	*p* = 0.042	7/46	*p* = 0.437
	No	29/37		26/37		4/37		8/37	
Chronic heart disease	Yes	32/41	*p* = 0.353	32/41	*p* = 0.330	9/41	*p* = 0.542	6/41	*p* = 0.447
	No	35/42		30/42		7/42		9/42	
Diabetes mellitus	Yes	27/28	*p* = 0.004	27/28	*p* = 0.031	8/28	*p* = 0.109	2/28	*p* = 0.096
	No	40/55		40/55		8/55		13/55	
Chronic kidney disease	Yes	11/13	*p* = 0.227	10/13	*p* = 0.574	5/13	*p* = 0.046	4/13	*p* = 0.050
	No	56/70		52/70		11/70		11/70	
Chronic liver disease	Yes	5/7	*p* = 0.289	5/7	*p* = 0.571	3/7	*p* = 0.098	2/7	*p* = 0.125
	No	62/76		57/76		13/76		13/76	
Malignant neoplasm	Yes	5/5	*p* = 0.450	5/5	*p* = 0.223	0/5	*p* = 0.260	0/5	*p* = 0.242
	No	62/78		57/78		16/78		15/78	
Taking anticoagulants	Yes	22/30	*p* = 0.417	31/30	*p* = 0.313	7/30	*p* = 0.566	3/30	*p* = 0.225
	No	45/53		41/53		9/53		12/53	
Taking antiaggregants	Yes	26/34	*p* = 0.790	26/34	*p* = 0.350	7/34	*p* = 0.508	5/34	*p* = 0.408
	No	39/49		36/48		9/49		10/49	
Taking NSAIDs	Yes	23/25	*p* = 0.049	23/25	*p* = 0.047	4/25	*p* = 0.766	7/25	*p* = 0.136
	No	44/58		39/57		12/58		8/58	
INR > 2	Yes	45/50	*p* = 0.134	42/50	*p* = 0.173	11/50	*p* = 0.186	10/50	*p* = 0.943
	No	48/58		46/58		9/58		11/58	
Heat rate/SBP ratio < 1	Yes	14/17	*p* = 0.724	12/17	*p* = 0.756	3/17	*p* = 0.848	6/17	*p* = 0.018
	No	53/66		50/66		13/66		9/66	
BUN/creatinine ratio > 30	Yes	36/42	*p* = 0.328	32/42	*p* = 0.807	10/42	*p* = 0.289	8/42	*p* = 0.829
	No	31/41		30/41		6/41		7/41	
Altered mental status	Yes	19/25	*p* = 0.574	18/25	*p* = 0.796	5/25	*p* = 0.913	4/25	*p* = 0.943
	No	48/58		44/58		11/58		11/58	
Need for blood transfusion	Yes	42/51	*p* = 0.865	39/51	*p* = 0.794	11/51	*p* = 0.504	9/51	*p* = 0.803
	No	25/32		23/32		5/32		6/32	
Complete SB examination	Yes	53/68	*p* = 0.584	49/68	*p* = 0.100	15/68	*p* = 0.281	13/68	*p* = 0.634
	No	12/15		13/15		1/15		2/15	
Adequate SB cleansing	Yes	53/66	*p* = 0.509	50/66	*p* = 0.756	15/66	*p* = 0.172	14/66	*p* = 0.274
	No	12/17		12/17		1/17		1/17	

Blood urea nitrogen (BUN); non-steroidal anti-inflammatory drugs (NSAIDs); number (n); small bowel (SB); systolic blood pressure (SBP).

**Table 3:** Final models with predictive factors for the detection of positive findings, need for endoscopic or surgical therapy, and rebleeding (multivariable analysis).

	Multivariable Models
**Positive findings on SBCE**	Intercept: B = 1.529, *p* = 0.001 NSAIDs: B = 1.248, *p* = 0.089, OR = 3.484 (95%CI 0.961–5.119) Diabetes mellitus: B = 2.353, *p* = 0.002, OR = 8.817 (95%CI 6.850–10.990) Nagelkerke’s R squared = 0.233
**Findings in the SB**	Intercept: B = 1.156, *p* = 0.050 NSAIDs: B = 0.506, *p* = 0.450, OR = 1.658 (95%CI 0.720–2.439) Diabetes mellitus: B = 2.686, *p* = 0.012, OR = 10.666 (95%CI 6.401–13.804) Nagelkerke’s R squared = 0.225
**Vascular lesion**	Intercept: B = 0.341, *p* = 0.634 Chronic kidney disease: B = 1.598, *p* = 0.029, OR = 4.944 (95%CI 2.901–6.889) Presenting with melena: B = 1.388, *p* = 0.044, OR = 4.007 (95%CI 1.981–6.372) Nagelkerke’s R squared = 0.259
**Findings outside the SB**	Intercept: B = −0.255, *p* = 0.712 Chronic kidney disease: B = 0.880, *p* = 0.199, OR = 2.410 (95%CI 0.918–3.921) Heat rate/SBP ratio <1 at admission: B = 1.234, *p* = 0.045, OR = 3.436 (95%CI 1.119–5.403) Nagelkerke’s R squared = 0.168
**Endoscopic, surgical, or angiographic therapy**	Intercept: B = 0.0.80, *p* = 0.777 Male: B = −1.061, *p* = 0.028, OR = 0.346 (95%CI 0.188–0.679) Nagelkerke’s R squared = 0.090
**Rebleeding**	Intercept: B = 1.735, *p* = 0.003 Antiaggregant drugs: B = 0.737, *p* = 0.254, OR = 2.090 (95%CI 0.870–4.441) Anticoagulants: B = 1.358, *p* = 0.033, OR = 3.886 (95%CI 2.452–4.871) Male: B = −1.951, *p* = 0.005, OR = 0.142 (95%CI 0.100–0.249) Nagelkerke’s R squared = 0.298

Blood urea nitrogen (BUN); confidence interval (CI); odds ratio (OR); non-steroidal anti-inflammatory drugs (NSAIDs); small bowel (SB); systolic blood pressure (SBP).

**Table 4:** Comparison of characteristics among patients submitted to endoscopic or surgical therapy and presenting rebleeding (univariate analysis).

Baseline Characteristics		Endoscopic, Surgical, or Angiographic Therapy (n = 48)			Rebleeding (n = 17)		
		Proportion	Percentage	*p*-Value	Proportion	Percentage	*p*-Value
Male sex	Yes	24/50	48.0	0.026	4/50	8.0	<0.001
	No	24/33	72.7		13/33	39.4	
Presenting with melena	Yes	27/46	58.7	0.859	10/46	21.7	0.752
	No	21/37	56.8		7/37	18.9	
Chronic heart disease	Yes	23/41	56.1	0.752	7/41	17.1	0.447
	No	25/42	59.5		10/42	23.8	
Diabetes mellitus	Yes	18/28	64.3	0.271	10/55	18.2	0.567
	No	30/55	54.5		7/28	25.0	
Chronic kidney disease	Yes	9/13	69.2	0.365	4/13	30.8	0.317
	No	39/70	55.7		13/70	18.6	
Chronic liver disease	Yes	3/7	42.9	0.402	1/7	14.3	0.671
	No	45/76	59.2		16/76	21.1	
Malignant neoplasm	Yes	3/5	60.0	0.919	2/5	40.0	0.265
	No	45/78	57.7		15/78	19.2	
Taking anticoagulants	Yes	13/30	43.3	0.144	10/30	33.3	0.029
	No	25/53	47.2		7/53	13.2	
Taking antiaggregants	Yes	15/34	44.1	0.470	11/34	32.4	0.031
	No	20/49	40.8		6/49	12.2	
Taking NSAIDs	Yes	17/24	70.8	0.142	18/24	75.0	0.555
	No	31/59	52.5		41/59	69.5	
INR > 2	Yes	13/25	52.0	0.134	5/25	20.0	0.943
	No	35/58	60.3		12/58	20.7	
Heat rate/SBP ratio < 1	Yes	10/17	58.8	0.926	2/17	11.8	0.318
	No	38/66	57.6		15/66	22.7	
BUN/creatinine ratio > 30	Yes	27/42	64.3	0.228	11/42	26.2	0.192
	No	21/41	51.2		6/41	14.6	
Altered mental status	Yes	13/25	52.0	0.480	4/25	16.0	0.507
	No	35/58	60.3		13/58	22.4	
Need for blood transfusion	Yes	32/51	62.7	0.252	13/51	25.5	0.153
	No	16/32	50.0		4/32	12.5	
Complete SB examination	Yes	39/68	57.4	0.944	15/68	22.1	0.725
	No	9/15	60.0		2/15	23.3	
Adequate SB cleansing (≥7 on Brotz scale)	Yes	35/66	53.0	0.102	12/66	18.2	0.324
	No	13/17	76.5		5/17	29.4	

Blood urea nitrogen (BUN); international normalized ratio (INR); non-steroidal anti-inflammatory drugs (NSAIDs); systolic blood pressure (SBP).

## Data Availability

The data will be shared upon reasonable request to the corresponding author.

## Supplementary Materials

The following supporting information can be downloaded at: https://www.mdpi.com/article/10.3390/diagnostics12112685/s1, Table S1. Capsule models used in the study, numbers of patients, and incomplete examinations. Table S2. Influence of patient-related factors on SBCE completeness and bowel cleansing.
