# Supplementary material for: Very High Yield of Urgent Small-Bowel Capsule Endoscopy for Ongoing Overt Suspected Small-Bowel Bleeding Irrespective of the Usual Predictive Factors
Source: Diagnostics (Basel). 2022 Nov 4;12(11):2685. doi: 10.3390/diagnostics12112685 (PMC9689099; doi:10.3390/diagnostics12112685)
Supplement: Supplementary file 1 [file diagnostics-12-02685-s001.zip › diagnostics-1977722-supplementary.pdf]

Supplementary table S1. Capsule models used in the study, numbers of patients and incomplete examinations.

Capsule model	Number of patients	Number of incomplete examinations
Mirocam MC1200	19	5
Mirocam MC1600	47	7
Pillcam SB	5	2
Pillcam SB2	12	1
Total	83	15

Supplementary table S2. Influence of patient-related factors on SBCE completeness and bowel cleansing.

Baseline characteristics		Incomplete SBCE (n=15)		Inadequate cleansing (n=17)	
Male sex	Yes	11/50	p=0.383	9/50	p=0.582
	No	4/33		8/33	
Age equal or above to 65 years old	Yes	9/49	p=0.886	11/49	**p=0.046**
	No	6/34		6/34	
Chronic heart disease	Yes	9/41	p=0.267	10/41	p=0.426
	No	6/42		7/42	
Diabetes mellitus	Yes	9/28	**p=0.032**	4/28	p=0.397
	No	6/55		13/55	
Chronic kidney disease	Yes	2/13	p=0.570	1/13	p=0.286
	No	13/70		16/70	
Chronic liver disease	Yes	2/7	p=0.605	1/7	p=0.998
	No	13/76		16/76	
